# The risk and risk factors of chikungunya virus infection and rheumatological sequelae in a cohort of U.S. Military Health System beneficiaries: Implications for the vaccine era

**DOI:** 10.1371/journal.pntd.0011810

**Published:** 2024-08-05

**Authors:** Simon Pollett, Hsing-Chuan Hsieh, Dan Lu, Melissa Grance, Stephanie Richard, Gosia Nowak, Charlotte Lanteri, David Tribble, Timothy Burgess

**Affiliations:** 1 Infectious Disease Clinical Research Program, Department of Preventive Medicine and Biostatistics, Uniformed Services University of the Health Sciences, Bethesda, Maryland, United States of America; 2 Henry M. Jackson Foundation for the Advancement of Military Medicine, Inc., Bethesda, Maryland, United States of America; 3 EpiData Center, Navy and Marine Corps Public Health Center, Portsmouth, Virginia, United States of America; Broad Institute Harvard: Broad Institute, UNITED STATES OF AMERICA

## Abstract

**Background:**

Understanding the risk of chikungunya virus (CHIKV) infection and rheumatic sequelae across populations, including travelers and the military, is critical. We leveraged healthcare delivery data of over 9 million U.S. Military Health System (MHS) beneficiaries to identify cases, and sampled controls, to estimate the risk of post-CHIKV rheumatic sequelae.

**Methodology/principal findings:**

MHS beneficiary CHIKV infections diagnosed 2014–2018 were identified from the Disease Reporting System internet, TRICARE Encounter Data Non-Institutional, and Comprehensive Ambulatory/Professional Encounter Record systems. Non-CHIKV controls were matched (1:4) by age, gender, beneficiary status, and encounter date. The frequency of comorbidities and incident rheumatic diagnoses through December 2018 were derived from International Classification of Diseases codes and compared between cases and controls. Poisson regression models estimated the association of CHIKV infection with rheumatic sequelae. We further performed a nested case-control study to estimate risk factors for post-CHIKV sequelae in those with prior CHIKV. 195 CHIKV cases were diagnosed between July 2014 and December 2018. The median age was 42 years, and 43.6% were active duty. 63/195 (32.3%) of CHIKV cases had an incident rheumatic diagnosis, including arthralgia, polyarthritis, polymyalgia rheumatica, and/or rheumatoid arthritis, compared to 156/780 (20.0%) of controls (p < 0.001). CHIKV infection remained associated with rheumatic sequelae (aRR = 1.579, p = 0.008) after adjusting for prior rheumatic disease and demography. Those with rheumatic CHIKV sequelae had a median 7 healthcare encounters (IQR 3–15). Among CHIKV infections, we found no association between post-CHIKV rheumatic sequelae and demography, service characteristics, or comorbidities.

**Conclusions/significance:**

CHIKV infection is uncommon but associated with rheumatic sequelae among MHS beneficiaries, with substantial healthcare requirements in a proportion of cases with such sequelae. No demographic, clinical, or occupational variables were associated with post-CHIKV rheumatic sequelae, suggesting that prediction of these complications is challenging in MHS beneficiaries. These findings are important context for future CHIKV vaccine decision making in this and other populations.

## Author summary

We examined U.S. Military Health System (MHS) healthcare delivery data to identify the likelihood of rheumatic complications after chikungunya virus (CHIKV) infection. Overall, CHIKV infections were rare in the MHS, with 195 cases found in the records between 2014 and 2018 (a period which encompassed the peak of the CHIKV epidemic in the Americas). Of these, about 32% received a rheumatic diagnosis after infection, including arthralgia, polyarthritis, polymyalgia rheumatica, and rheumatoid arthritis. Patients who had a rheumatic diagnosis had on average 7 healthcare encounters for their post-CHIKV rheumatic complication, and a quarter had more than 15 healthcare encounters. We did not find any demographic, clinical, or occupational characteristics associated with developing rheumatic complications after CHIKV, suggesting that predicting rheumatic complications from CHIKV may be challenging in MHS beneficiaries. These findings may provide important context for decisions about implementing an approved chikungunya vaccine to military servicemembers and other MHS beneficiaries.

## Introduction

Chikungunya virus (CHIKV) is a mosquito-borne alphavirus belonging to the *Togaviridae* family [1]. The ecology of CHIKV is characterized by a sylvatic cycle involving non-human primates and arboreal *Aedes* species and urban human-to-human transmission cycles involving the *A*. *aegypti* and *A*. *albopictus* vectors [1]. While CHIKV is defined by a single serotype, multiple genotypes have evolved with global geographic expansion including the Asian and descendant Asian-Caribbean lineages, and East/Central/South African and Indian Ocean lineages [1–3].

The 2013–2015 neotropical epidemic in the Americas involved over 1.6 million suspected or confirmed CHIKV cases [4]. While cases rapidly declined after the initial neotropical pandemic, there is an ongoing substantive CHIKV burden in the Americas in addition to ongoing circulation in Africa [5,6] and Asia [7,8]. Recently, the Pan American Health Organization issued an alert indicating the geographic range of CHIKV has expanded in the Americas, with notable resurgence in certain regions of Latin America [9–11].

Acute CHIKV infection illness is characterized by a high symptomatic-to-asymptomatic ratio, with clinical features including an abrupt onset of high fever, polyarthralgia, backache, headache, and fatigue [12]. Atypical acute CHIKV illness manifestations (e.g., acute encephalitis) are rare, potentially severe, and more often occur in the extremes of age and in those with underlying comorbidities [12–17]. Diagnosis of CHIKV infection relies on blood polymerase chain reaction (PCR) within the first week of illness and serology thereafter [18]. Management of acute CHIKV is generally supportive, with fluid replacement, analgesia and avoidance of non-steroidal anti-inflammatory drugs until dengue has been excluded as a differential diagnosis [19]. There are limited preventive medical countermeasures available for CHIKV aside from avoidance of vectors in endemic transmission regions [19]. Advanced vaccine development has been challenging due to the unpredictable nature of CHIKV outbreaks [20]. However, CHIKV vaccine U.S. Food and Drug Administration licensure recently occurred using a serological correlate of protection, and other vaccines may be licensed in the future [21].

The rheumatological manifestations of CHIKV may be categorized by timing and clinical phenotype [22]. Acute CHIKV arthralgia is frequently reported and mostly occurs in mostly peripheral joints with a symmetrical pattern, although large joints can be affected [22]. Joint swelling and synovitis often accompanies this acute polyarthralgia; functional impairment during this acute phase is common due debilitating pain [22]. Chronic rheumatological manifestations typically involve the joints affected in acute CHIKV arthralgia [22]. Estimates on the frequency of persistent post-acute manifestations (predominantly arthralgia or arthritis) vary significantly, with metanalysis estimates as high as 43% at 3 months, 21% at 12 months, and 14% at 18 months after initial CHIKV illness onset [22–24]. Pathogenesis studies have suggested that persistent host inflammation, rather than persistent synovial viral replication, plays a key role in post-CHIKV arthralgia [25,26]. There is currently a wide variation in clinical management for chronic post-CHIKV sequelae [15] but post-acute CHIKV arthropathy is severe enough in some to warrant the use of biological or disease-modifying antirheumatic drugs [27].

While those residing in CHIKV endemic regions experience the highest burden of this arbovirus, travelers and the deploying military are also significant stakeholders for understanding the risk of CHIKV infection and post-CHIKV sequelae. There is limited published data on the burden of CHIKV in U.S. Military Health System (MHS) beneficiaries [28,29] and none define the risk and risk factors of post-acute CHIKV sequelae in this population. Understanding the risk and risk factors of CHIKV and CHIKV sequelae is important for clinical prognostication and may support cost-effectiveness analyses for vaccine policy with current and potential future licensed vaccines; such findings may be generalizable to traveler populations also.

The objectives in this study were to characterize incident CHIKV cases diagnosed in the MHS between 2005 and 2018 and to describe the risk and risk factors of acute and chronic post-CHIKV rheumatological sequelae in CHIKV cases compared to matched controls. Here, we leveraged a virtual cohort derived from over 9.5 million MHS beneficiaries, with follow-up through December 2018.

## Methods

### Ethics statement

This protocol was reviewed and approved for execution by the Institutional Review Board of the Uniformed Services University of the Health Sciences, Bethesda, Maryland, United States of America.

### Population, setting and identification of CHIKV infections and matched controls

The MHS serves approximately 9.5 million beneficiaries, including active duty servicemembers, retirees and dependents. This includes care delivered within the United States, U.S. Territories (including Puerto Rico, Guam, and the U.S. Virgin Islands), and in beneficiaries located other countries.

For this study, we identified deduplicated unique CHIKV cases in MHS beneficiaries via multiple data streams from 2005 through 2018, including: (i) clinical microbiology lab (serology and PCR) results from Disease Reporting System internet (DRSi)/EpiData Center, (ii) International Classification of Diseases, 10^th^ Revision (ICD-10) code A92.0 CHIKV disease entries from TRICARE Encounter Data Non-Institutional (TEDNI), and (iii) ICD-10 code A92.0 CHIKV disease from Comprehensive Ambulatory/Professional Encounter Record (CAPER). The treatment locations of cases were inferred by clinical provider ZIP code. To estimate the risk of post-CHIKV rheumatological sequalae, CHIKV negative MHS beneficiary controls were matched (4:1) with CHIKV positive beneficiaries by age group, gender, date of healthcare encounter (+/- 1 month), and beneficiary status at the time of matching healthcare encounter. Controls were randomly selected within these matching strata.

### Demographic, post-CHIKV diagnosis and comorbidity data abstraction from the electronic medical record

Demographic ICD-9 and ICD-10 code data were abstracted from cases and controls (using Armed Forces Health Longitudinal Technology Application (AHLTA) and the Composite Health Care System (CHCS)). The sequelae of interest were any rheumatologic disorders (Table A in S1 Text for ICD-9 and ICD-10 codes). Co-morbidity was defined as a condition/disorder (including rheumatological) which pre-existed before the time of the first CHIKV infection diagnosis (or first matched healthcare encounter of the time-matched control). We defined post-CHIKV rheumatological complications as new post-CHIKV rheumatological diagnoses with a first onset at or after the first CHIKV diagnosis (or the healthcare encounter used for matching for controls without CHIKV). Due to the typically rapid onset of arthritis in CHIKV, and because diagnosis of CHIKV may occur upon presentation to clinical care with subacute or chronic rheumatological symptoms, we did not include a latency period between CHIKV diagnosis date and date of rheumatological diagnoses.

When a pre-CHIKV rheumatological and post-CHIKV rheumatological diagnosis mapped to the same ICD code, we further adjudicated a pre-existing rheumatological diagnosis from a new post-CHIKV complication using DX_DESC-level diagnosis coding (Agency for Healthcare Research and Quality Clinical Classifications Software system code). A similar approach was taken for controls without CHIKV.

### Statistical analysis

Demographic, clinical, and occupational characteristics of cases and controls were summarized. The frequency of rheumatological sequelae were compared in those with CHIKV infection (“cases”) and without CHIKV (“controls”). Multivariate Poisson regression on matched MHS beneficiaries with CHIKV infection and without CHIKV infection was used to estimate the association between CHIKV infection and rheumatological sequelae, with further analysis estimating the risk of CHIKV infection on short-term (<3 months) versus long-term (≥3 months) rheumatological complications, adjusting for pre-existing rheumatological comorbidities and demography. Unadjusted and adjusted risk ratios were presented. Finally, among those with CHIKV infections, we performed a nested case-control study comparing those with and without rheumatological sequelae in order to identify risk factors for developing post-CHIKV rheumatological sequelae. Unadjusted and adjusted odds ratios were presented.

All p values were two-sided, with an alpha significance threshold of 0.05. Analyses were conducted using SAS software (version 9.4; SAS).

## Results

**Fig 1:**
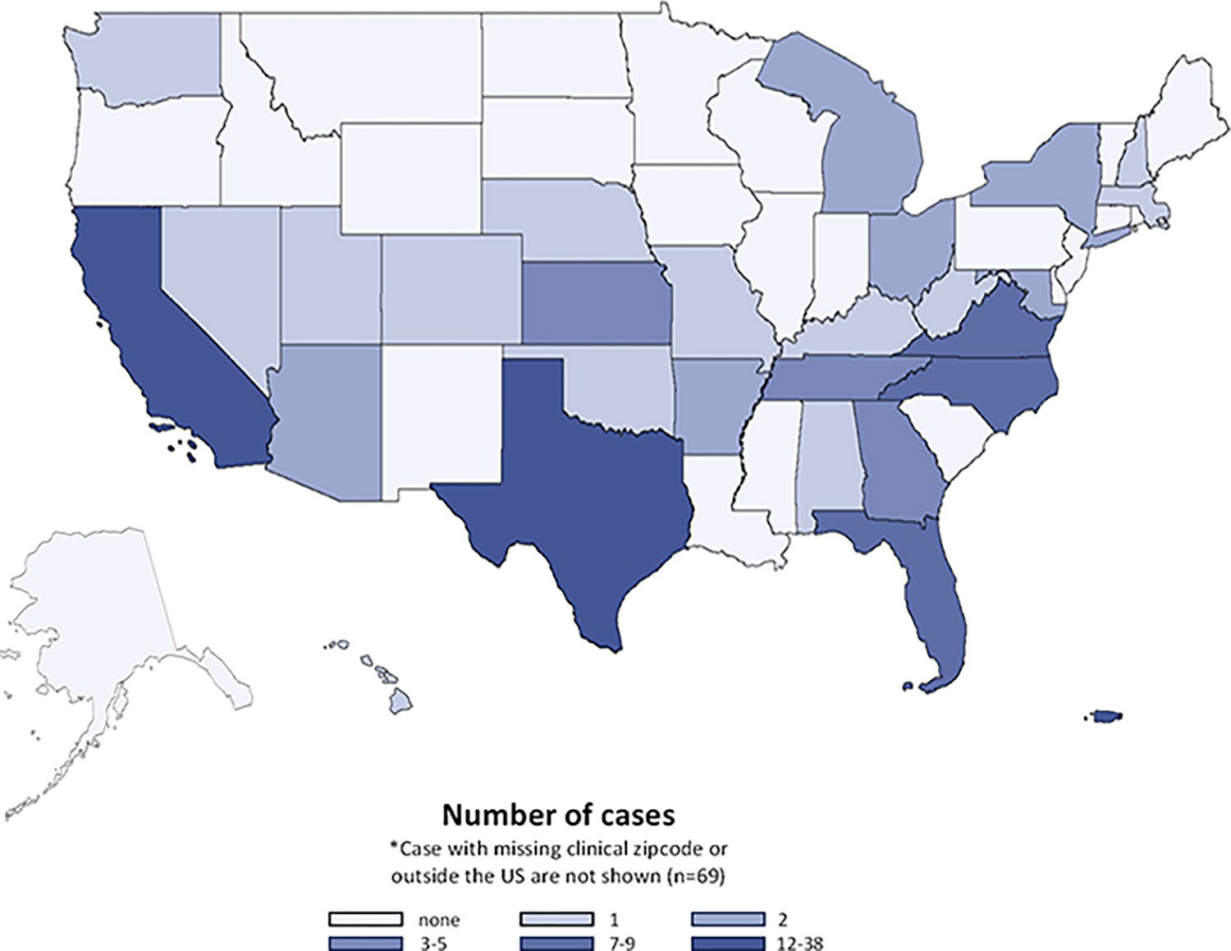
Location of CHIKV cases by U.S. state or territory. Map rendered by SAS using the ‘2023 TIGER/Line Shapefiles: States (and equivalent)’ with the public domain basemap provided by United States Census Bureau; source: US Census Bureau, Geography Division.

We identified 195 unique CHIKV cases diagnosed in the MHS between July 2014 and December 2018 (Fig A in S1 Text). The most frequent locations of clinical encounters were in Puerto Rico, Texas, and California, but these reflect provider ZIP codes of diagnoses (including potential diagnoses made after deployments or other beneficiary travel) and do not necessarily reflect the location where the cases acquired CHIKV infections (Fig 1). Our finding of the majority of cases being diagnosed in Puerto Rico was similar to prior studies in MHS beneficiaries [29,30]. Time series of all MHS CHIKV cases (by month) indicated the peak of MHS CHIKV cases occurred in late 2014 during the peak year of the Americas CHIKV epidemic.

The median age of CHIKV cases was 42 years (IQR 31–54 years), 52.8% were male, and 43.6% were active duty servicemembers (Table 1). Compared to age, gender, beneficiary status, and healthcare encounter-matched controls, cases differed from controls with respect to race (p < 0.001). White, Asian/Pacific Islander, and Other race categories were more common in CHIKV cases compared to controls. Among active duty CHIKV cases, we noted that Army service and enlisted rank were most common; the frequency of these categories were more common than matched controls (p < 0.001; Table 1). CHIKV cases had a higher frequency of all measured comorbidities compared to matched controls (p < 0.001; Table 2).

**Table 1:** Demographic and professional characteristics in CHIKV cases and controls.

	CHIKV negative controls^a^	CHIKV cases	Total	P value^b^
**No. of subjects (N)**	780	195	975	
**Characteristic**	n (n/N %)	n (n/N %)	n (n/N %)	
**Gender**				>.9999^a^
Male	412 (52.82)	103 (52.82)	515 (52.82)	
Female	368 (47.18)	92 (47.18)	460 (47.18)	
**Age,** median, [Q1–Q3]	41 [29–56]	42 [31–54]	41 [30–55]	0.6026
**Age**				>.9999^a^
≤25	84 (10.77)	21 (10.77)	105 (10.77)	
26–35	204 (26.15)	51 (26.15)	255 (26.15)	
36–45	190 (24.36)	48 (24.62)	238 (24.41)	
46–64	204 (26.15)	51 (26.15)	255 (26.15)	
65+	98 (12.56)	24 (12.31)	122 (12.51)	
**Race**				< .0001
White	321 (41.15)	113 (57.95)	434 (44.51)	
Black	55 (7.05)	12 (6.15)	67 (6.87)	
Asian or Pacific Islander	23 (2.95)	12 (6.15)	35 (3.59)	
Other	21 (2.69)	30 (15.38)	51 (5.23)	
Unknown	360 (46.15)	28 (14.36)	388 (39.79)	
**Beneficiary**				>.9999^a^
Active duty	340 (43.59)	85 (43.59)	425 (43.59)	
Dependent	108 (13.85)	27 (13.85)	135 (13.85)	
Retiree	80 (10.26)	20 (10.26)	100 (10.26)	
Others	252 (32.31)	63 (32.31)	315 (32.31)	
**Service branch** ^**c**^				< .0001
Air Force	189 (24.23)	8 (4.10)	197 (20.21)	
Army	76 (9.74)	51 (26.15)	127 (13.03)	
Coast Guard	8 (1.03)	23 (11.79)	31 (3.18)	
Other	67 (8.59)	3 (1.54)	70 (7.18)	
Not applicable	440 (56.41)	110 (56.41)	550 (56.41)	
**Rank** ^d^				< .0001
Enlisted	233 (29.87)	69 (35.38)	302 (30.97)	
Officer	107 (13.72)	16 (8.21)	123 (12.62)	
Not applicable	440 (56.41)	110 (56.41)	550 (56.41)	

^a^Controls were matched with cases by age group, gender, beneficiary status, and date of encounter (+/- 1 month).

^b^p-values are calculated from a Chi-squared/Fisher’s exact test or Kruskal-Wallis test, as appropriate.

^c^Service branch and rank are only available for active duty. Other non-active duty subjects are grouped under “Not applicable” category.

^d^Refer to https://www.defense.gov/Resources/Insignia/ for general definitions of enlisted versus officer.

**Table 2:** Comorbidities in CHIKV cases and controls.

	CHIKV-	CHIKV+	Total	P value^a^
**Total # of subjects**	780	195	975	
**Comorbidities (ICD diagnoses before time of matching)**				
None	461 (59.10)	34 (17.44)	495 (50.77)	< .0001
Cancer	30 (3.85)	22 (11.28)	52 (5.33)	< .0001
Cardiovascular disease	196 (25.13)	95 (48.72)	291 (29.85)	< .0001
Chronic respiratory disease	91 (11.67)	58 (29.74)	149 (15.28)	< .0001
Mental health condition	117 (15.00)	72 (36.92)	189 (19.38)	< .0001
Osteoporosis	26 (3.33)	19 (9.74)	45 (4.62)	0.0001
Upper gastrointestinal disease	103 (13.21)	75 (38.46)	178 (18.26)	< .0001
Neurologic disease	122 (15.64)	80 (41.03)	202 (20.72)	< .0001
Rheumatologic disease	117 (15.00)	84 (43.08)	201 (20.62)	< .0001
Osteoarthritis	92 (11.79)	55 (28.21)	147 (15.08)	< .0001
Other non-traumatic joint disorders	29 (3.72)	32 (16.41)	61 (6.26)	< .0001
Rheumatoid arthritis and related disease	12 (1.54)	17 (8.72)	29 (2.97)	< .0001
Spondylosis; disc disorders; other back problems	51 (6.54)	37 (18.97)	88 (9.03)	< .0001

^a^p values were calculated from a Chi-squared/Fisher’s exact test.

We noted 63/195 (32.3%) of CHIKV cases had an incident post-CHIKV rheumatological diagnosis, compared to 156/780 (20.0%) of controls (p = 0.0002) (Table 3). Osteoarthritis, rheumatoid arthritis and related diagnoses (including inflammatory polyarthropathy), and non-traumatic joint disorder associated categories (including unspecified arthropathy, polyarthralgia and polyarthritis) were noted with statistically significant higher frequency in post-CHIKV cases versus controls (Tables 3 and B in S1 Text).

**Table 3:** Incident rheumatological diagnoses in CHIKV cases and controls.

	CHIK-	CHIK+	Total	P value^a^
**No. of subjects**	780	195	975	
**Any incident rheumatological diagnosis** ^**b**^	156 (20.00)	63 (32.31)	219 (22.46)	0.0002
**Incident rheumatological diagnosis by category** ^**c**^				
Infective arthritis and osteomyelitis	0 (0.00)	1 (0.51)	1 (0.10)	0.2000
Malaise and fatigue	59 (7.56)	18 (9.23)	77 (7.90)	0.4402
Osteoarthritis	2 (0.26)	4 (2.05)	6 (0.62)	0.0166
Other connective tissue disease	12 (1.54)	4 (2.05)	16 (1.64)	0.5404
Other non-traumatic joint disorders	17 (2.18)	21 (10.77)	38 (3.90)	< .0001
Other screening for suspected conditions	81 (10.38)	24 (12.31)	105 (10.77)	0.4384
Rheumatoid arthritis and related disease	2 (0.26)	7 (3.59)	9 (0.92)	0.0003

^a^p values were calculated from a Chi-squared/Fisher’s exact test.

^b^Subject with more than one diagnosis code were only counted once.

^c^CCS_DX_DESC category, subject may have multiple diagnosis with different categories.

Compared to controls, CHIKV infection was associated with post-infection rheumatological diagnoses (aRR = 1.579, 95% CI 1.125–2.215, p = 0.008) after adjusting for the presence of any prior pre-CHIKV rheumatological diagnoses, service status, age, sex, and race (Table 4). The magnitude of this effect size was larger when the outcome was stratified into short-term (≤3 month) post-CHIKV rheumatological diagnoses versus long-term (diagnosis recurred beyond 3 months post-CHIKV infection) rheumatic sequelae (aRR = 4.093, p = 0.008, short-term sequelae; aRR = 1.482, p = 0.021 long-term sequelae, respectively) (Table 5). Those with post-CHIKV rheumatological incident diagnoses had a median 7 healthcare encounters for rheumatological diagnoses (IQR 3–15, range 1–33) through the period of observation.

**Table 4:** Unadjusted and adjusted risk ratio of CHIKV infection on rheumatic-related incident diagnoses (n = 975).

	Unadjusted				Adjusted			
	RR	95% CI		P-value	RR	95% CI		P-value
**CHIKV positive**	1.615	1.206	2.164	0.0013	1.579	1.125	2.215	0.0082
**Pre-existing rheumatic related comorbidity**	1.429	1.060	1.926	0.0191	1.090	0.777	1.528	0.6178
**Age**	1.008	1.001	1.015	0.0243	1.009	1.001	1.018	0.0373
**Male**	0.988	0.758	1.288	0.9270	0.935	0.668	1.307	0.6931
**Race (ref: White)**								
Black	1.047	0.617	1.775	0.8650	1.135	0.665	1.936	0.6434
Asian or Pacific Islander	0.877	0.407	1.887	0.7367	0.822	0.380	1.776	0.6176
Other^a^	0.969	0.732	1.282	0.8235	1.199	0.835	1.720	0.3251
**Beneficiary (ref: Active Duty)** ^**b**^								
Dependents of Active Duty	0.655	0.409	1.047	0.0772	**-**	**-**	**-**	**-**
Retirees	0.800	0.490	1.305	0.3709	**-**	**-**	**-**	**-**
Others	1.042	0.775	1.400	0.7851	**-**	**-**	**-**	**-**
**Service branch (ref: Air Force)**								
Army	1.703	1.115	2.599	0.0137	1.367	0.872	2.145	0.1730
Coast Guard	0.620	0.222	1.731	0.3614	0.456	0.158	1.316	0.1464
Other^c^	0.755	0.388	1.469	0.4080	0.739	0.378	1.445	0.3771
Not applicable (non-active duty)	1.031	0.723	1.471	0.8668	0.718	0.454	1.135	0.1558
**Rank (ref: Enlisted)^b^**								
Officer	1.419	0.947	2.128	0.0899	**-**	**-**	**-**	**-**
Not applicable (non-active duty)	1.012	0.747	1.372	0.9368	**-**	**-**	**-**	**-**

^a^Other includes American Indian, Alaskan native, and all others

^b^Beneficiary and rank are not included in the adjusted model due to collinearity with service branch

^c^Other includes Marines, Navy and all others

**Table 5:** Adjusted risk ratio of CHIKV infection on rheumatic-related short-term and long-term sequelae.

	Short-term (< 3 month) (n = 773)^a^				Long-term (≥ 3 month) (n = 958)^b^			
	RR	95% CI		P-value	RR	95% CI		P-value
**CHIKV positive**	4.093	1.449	11.562	0.0078	1.482	1.063	2.068	0.0205
**Pre-existing rheumatic related comorbidity**	0.453	0.114	1.806	0.2618	1.203	0.850	1.703	0.2979
**Age**	1.018	0.991	1.045	0.1984	1.006	0.998	1.015	0.1192
**Male**	1.509	0.494	4.604	0.4700	1.022	0.739	1.413	0.8964
**Race (ref: White)**								
** Black**	0.931	0.115	7.508	0.9461	1.069	0.617	1.854	0.8110
**Asian or Pacific Islander**^**c**^	**-**	**-**	**-**	**-**	0.923	0.427	1.994	0.8384
** Other**	1.381	0.439	4.341	0.5805	1.005	0.710	1.422	0.9786

^***a”***^*Short-term sequelae” was defined as those who had rheumatic related sequelae diagnosed within the first 3 months followed CHIKV infection or a matched initial encounter date for CHIKV-negative group (baseline) without having any diagnoses after 3 months post baseline*. *Individuals who had rheumatic related sequelae diagnosed after 3 months post baseline were excluded from the analysis*.

^***b”***^*Long term sequelae” was defined as those who had rheumatic related sequelae diagnosed after 3 months post baseline*. *Individuals who had rheumatic related sequelae that were only diagnosed within the first 3 months after baseline were excluded from the analysis*.

^***c***^*None of Asian or Pacific Islander MHS beneficiaries were observed as having only short-term rheumatic sequelae*.

Among those with CHIKV infections, we found no association between post-CHIKV rheumatic sequelae and age, sex, race, active-duty status, or pre-existing comorbidities. Service branch was associated with post-CHIKV rheumatological sequelae, but this was not statistically significant (Table 6).

**Table 6:** Unadjusted and adjusted odds ratio of rheumatic-related sequelae among people with CHIKV infection (n = 195).

	Unadjusted				Adjusted			
	OR	95% CI		p value	OR	95% CI		p value
**Age**	1.002	0.985	1.020	0.8156	1.006	0.978	1.034	0.6969
**Male (ref: female)**	0.974	0.534	1.777	0.9323	0.679	0.288	1.599	0.3751
**Race (ref: white)**								
Black	0.685	0.175	2.680	0.5865	0.624	0.138	2.816	0.4970
Asian or Pacific Islander	1.467	0.436	4.935	0.5356	1.350	0.376	4.853	0.4577
Other	0.924	0.468	1.826	0.8208	0.866	0.397	1.888	0.8440
**Service branch (ref: Army)**								
Coast Guard	0.256	0.076	0.860	0.0276	0.275	0.077	0.979	0.0917
Other	1.461	0.395	5.407	0.5703	1.566	0.406	6.042	0.0634
Not applicable (non-active duty)	0.457	0.228	0.913	0.0266	0.341	0.141	0.828	0.0922
**Comorbidities**								
Cancer	0.975	0.376	2.526	0.9586	1.029	0.324	3.269	0.9618
Cardiovascular disease	0.937	0.514	1.709	0.8320	0.799	0.371	1.720	0.5659
Chronic respiratory disease	1.431	0.751	2.725	0.2757	1.902	0.841	4.300	0.1226
Mental health condition	0.974	0.523	1.815	0.9339	0.889	0.420	1.881	0.7575
Osteoporosis	1.250	0.467	3.346	0.6569	1.488	0.390	5.680	0.5609
Upper gastrointestinal disease	0.977	0.527	1.812	0.9422	0.988	0.482	2.024	0.9737
Neurologic disease	0.757	0.408	1.403	0.3762	0.657	0.300	1.439	0.2941
Rheumatologic	1.086	0.593	1.988	0.7899	0.985	0.472	2.059	0.9688

*Note*: Service branch was regrouped to avoid quasi-complete separation. Other includes: Air Force and others.

## Discussion

Understanding the risk of CHIKV infection and complications in deploying U.S. military servicemembers and other MHS beneficiaries is important, particularly with recent and upcoming possible further CHIKV vaccine licensure. We found a very low number of CHIKV cases identified in the MHS, despite this study period covering the peak CHIKV epidemic in the Americas and leveraging data from about 9.5 million beneficiaries. Our findings correlate with low number of CHIKV infections in other U.S. MHS studies, although such studies were either smaller, earlier in the CHIKV epidemic and/or relied on varying electronic medical record (eMR) data sources [28,31]. Additionally, our findings correlate with a recent post-deployment serology study of 1500 deployments to South America and Puerto Rico with a low post-deployment seropositivity rate of 1.7% (highest in those serving in Puerto Rico) [32]. Our findings further agree with studies in other armed forces which also note a low frequency of CHIKV infections [28].

These low frequencies of infection may be attributed to effective personal mosquito avoidance or heterogeneity in risk exposure (for example, based on deployment role) when deployed or activated in CHIKV endemic countries or territories. Among CHIKV infections in servicemembers, we noted higher frequency Army service and enlisted ranks compared to matched servicemember controls without CHIKV; these groups have previously been noted at higher risk for other arboviruses [33,34]. While the location of diagnosis may not reflect where the infection was acquired, the highest number of cases were diagnosed in Puerto Rico is consistent with the known high transmission of CHIKV during the neotropical epidemic [35].

While the overall number of identified infections was very low, we found a substantive risk of post-CHIKV rheumatological complications. We noted a considerable burden of disease with a median 7 healthcare encounters for this complication, and 25% of these individuals had between 15 and 33 further encounters through the period of observation. These findings highlight that, while CHIKV infections are overall rare in MHS beneficiaries, patient morbidity in complicated CHIKV cases can be substantial, as noted in non-MHS studies [22].

The overall low number of CHIKV infections limited statistical power for examining risk factors for post-CHIKV sequelae. We found a non-statistically significant trend toward service branch having a differential risk of rheumatic complications, but no other predictors were noted, including pre-existing comorbidities. Further study of cumulative CHIKV infections in MHS-based virtual cohorts over time may identify clearer risk factors for this complication, and this may assist in more precise prognostication of CHIKV cases in the MHS. As this study comprises the largest number of CHIKV infections in any military-related population, we conclude that prediction of which MHS CHIKV cases may progress to complicated rheumatological sequelae remains challenging.

There were several strengths to this study. The use of the MHS includes a substantive number of beneficiaries who deploy or are stationed in CHIKV endemic regions and allowed for follow-up of cases to determine post-acute complications. We derived CHIKV cases from a range of eMR data sources, including clinical microbiology laboratory results, and analyzed a greater number of infections than prior MHS based studies [28–31]. Our study limitations included the use of ICD codes to diagnose CHIKV and post-CHIKV complications; prior evaluations of CHIKV ICD diagnosis coding have noted imperfect specificity [31] and this study was unable to perform chart adjudications. Conversely, CHIKV diagnoses may have been missed in some clinical presentations, and not all cases may have presented to care (including during deployments).

Future directions include studies to examine for non-rheumatological CHIKV sequelae such as neurocognitive complications, mood, and long-term functional impact (for example, [36,37]). Finally, studies could leverage sera routinely collected from the U.S. military servicemembers and used for arboviral research (i.e., the Department of Defense Sera Repository [32–34]) to identify prognostic and treatment related biomarkers for those beneficiaries with persistent post-CHIKV sequelae, as well as enabling ongoing serosurveillance of the risk and risk factors of CHIKV in deployed military, particularly during an era of increasing CHIKV risk in the Americas [9].

## Disclaimer

The contents of this publication are the sole responsibility of the author(s) and do not necessarily reflect the views, opinions, or policies of Uniformed Services University of the Health Sciences; the Department of Defense; the Departments of the Army, Navy, or Air Force; the Defense Health Agency; the National Institutes of Health, the Department of Health and Human Services or the Henry M. Jackson Foundation for the Advancement of Military Medicine, Inc. Mention of trade names, commercial products, or organizations does not imply endorsement by the U.S. government. The investigators have adhered to the policies for protection of human subjects as prescribed in 45 CFR 46.

## Supporting information

S1 Text Fig A. **Chikungunya virus case identification from U.S. Military Health System electronic medical record systems.** TEDNI–TRICARE Encounter Data Non-Institutional; CAPER–Comprehensive Ambulatory/Professional Encounter Record. **Table A. International Classification of Diseases, 9th Revision (ICD-9) and ICD-10 codes (including cross-walked ICD-9 codes) used to identify rheumatological healthcare encounters in cases and controls. Table B. Long description of rheumatological healthcare encounters by chikungunya virus (CHIKV) infection status.**
(DOCX)
